# Supplementary material for: Content-rich biological network constructed by mining PubMed abstracts
Source: BMC Bioinformatics. 2004 Oct 8;5:147. doi: 10.1186/1471-2105-5-147 (PMC528731; doi:10.1186/1471-2105-5-147)
Supplement: Additional File 2 — The original results of the above study (non-essential files are deleted to keep the file size under the limit set by BMC bioinformatics). [file 1471-2105-5-147-S2.bz2 › chilibotAdditionalFile2/dip30/236ID8006584E760/html/IL2RG_IL2.html]

 


 **IL2RG** and **IL2** 
  
Found 241 abstracts in PubMed, retrieved 30.  
 

 What does Google say? 
 PDF only 
| .edu only 

---

**Interactive relationship** (e.g. stimulation, inhibition, etc)

**Neutral relationship**- and binds to the 16 kDa subunit of the vacuolar ATPase pump, immature forms of the beta and gamma c  [ **IL2RG** ]  chains of the interleukin 2  [ **IL2** ]  receptor, and the free chain of MHC I. p12 carrying a lysine residue p12K at position 88 of its sequence may be rapidly degraded in the cell via proteasome.  Ref: 12230932 AIDS Res Hum Retroviruses, 2002
- Co transfection of IL 2Ralpha and IL 2Rbeta gamma c  [ **IL2RG** ]  chimeras diminished spontaneous IL 2Ralpha chimera oligomerization and permitted IL 2  [ **IL2** ]  inducible hetero oligomerization of receptor components.  Ref: 11886175 Cytokine, 2002
- Anti apoptotic signaling by the interleukin 2  [ **IL2** ]  receptor reveals a function for cytoplasmic tyrosine residues within the common gamma gamma c  [ **IL2RG** ]  receptor subunit.  Ref: 12525482 J Biol Chem, 2003
- Nonreceptor tyrosine kinase Jak3, which binds to intracellular domain of interleukin 2  [ **IL2** ]  receptor gamma  [ **IL2RG** ]  subunit IL 2Rgamma, plays an important role in IL 2  [ **IL2** ]  function.  Ref: 12136181 Sheng Wu Hua Xue Yu Sheng Wu Wu Li Xue Bao Shanghai < MedlineTA>Sheng Wu Hua Xue Yu Sheng Wu Wu Li Xue Bao Shanghai , 2003
- The receptor consists of two chains IL 7 specific alpha chain CD127 and the common gamma c  [ **IL2RG** ]  chain CD132  [ **IL2RG** ]  which is a component of several other cytokine receptors IL 2  [ **IL2** ] , IL 4, IL 9 and IL 15.  Ref: 12644318 Immunol Lett, 2003
- Previously it was known that the IL 2  [ **IL2** ]  and IL 7 receptors shared the common cytokine receptor gamma chain, gamma c  [ **IL2RG** ] , which suggested a possible competition between these cytokines for a receptor component.  Ref: 12354940 Proc Natl Acad Sci U S A, 2002
- The receptors for interleukin 2  [ **IL2** ]  IL 2  [ **IL2** ]  and interleukin 15 IL 15 in T cells share the IL 2R beta subunit CD122 and gamma C  [ **IL2RG** ]  subunit but have private alpha subunits.  Ref: 12121224 Immunol Cell Biol, 2002
- Our laboratory has identified an X linked severe combined immunodeficiency XSCID  [ **IL2RG** ]  in dogs that is the result of mutations in the common gamma chain gammac subunit of the interleukin 2  [ **IL2** ]  IL 2  [ **IL2** ] , IL 4, IL 7, IL 9, IL 15, and IL 21 receptors.  Ref: 12857971 Immunol Res, 2003
- The cytokine interleukin 21 IL 21 is closely related to IL 2  [ **IL2** ]  and IL 15, and their receptors all share the common cytokine receptor gamma chain, gammac, which is mutated in humans with X linked severe combined immunodeficiency disease XSCID  [ **IL2RG** ] .  Ref: 12446913 Science, 2002
- Interleukin IL 2  [ **IL2** ] , IL 4, IL 7, IL 9, IL 15, and IL 21 form a family of cytokines based on their sharing the common cytokine receptor gamma chain, gamma c  [ **IL2RG** ] , which is mutated in X linked severe combined immunodeficiency SCID.  Ref: 12435740 J Biol Chem, 2003
- Interleukin IL 2  [ **IL2** ]  receptor subunit alpha IL 2Ralpha increases the affinity of the IL 2  [ **IL2** ]  receptor complex while hetero association of IL 2Rbeta and gamma c  [ **IL2RG** ]  chains initiates a proliferative signal.  Ref: 11886175 Cytokine, 2002

**Non-interactive relationship** (e.g. studied together, co-existance, homology, etc.)

- Singly or co transfected IL 2Rbeta and gamma c  [ **IL2RG** ]  chimeras showed no spontaneous or IL 2  [ **IL2** ]  inducible oligomerization.  Ref: 11886175 Cytokine, 2002
- The common cytokine receptor gamma chain gamma c  [ **IL2RG** ] , an essential component of the receptors for IL 2  [ **IL2** ] , IL 4, IL 7, IL 9, and IL 15, is critical for the development and function of lymphocytes.  Ref: 12093291 Biochemistry, 2002
- Inhibition of endocytosis by genistein and overexpression of interleukin 2  [ **IL2** ]  receptor gamma  [ **IL2RG** ]  abolished the effect of biotin.  Ref: 12388078 Am J Physiol Cell Physiol, 2003
- Analysis of the role of the interleukin 2  [ **IL2** ]  receptor gamma chain  [ **IL2RG** ]  in ligand binding.  Ref: 11851401 Biochemistry, 2002
- IL 21R shares the common gamma chain gamma c  [ **IL2RG** ]  with the receptors for IL 2  [ **IL2** ] , IL 4, IL 7, IL 9, and IL 15.  Ref: 12244150 J Immunol, 2002
- Like living Trypanosoma cruzi, its AGC10 membrane glycoprotein inhibits interleukin 2  [ **IL2** ]  IL 2  [ **IL2** ]  secretion and membrane expression of CD25, CD122, and CD132  [ **IL2RG** ]  the components of the high affinity IL 2  [ **IL2** ]  receptor by activated human lymphocytes.  Ref: 12467977 Mol Biochem Parasitol< MedlineTA>Mol Biochem Parasitol, 2002
- Interleukin 2  [ **IL2** ]  receptor gamma chain  [ **IL2RG** ]  gamma c  [ **IL2RG** ]  knockout mice show less regularity in estrous cycle but achieve normal pregnancy without fetal compromise.  Ref: 12069389 Am J Reprod Immunol, 2002
- Interleukin 2  [ **IL2** ]  receptor gamma  [ **IL2RG** ]  dependent endocytosis depends on biotin in Jurkat cells.  Ref: 12388078 Am J Physiol Cell Physiol, 2003
- We studied the effect of cytokines on reproductive functions such as ovulation and pregnancy, with mutant mice lacking interleukin 2  [ **IL2** ]  receptor gamma  [ **IL2RG** ]  IL 2Rgamma, the so called common gamma chain gamma c  [ **IL2RG** ] , which is shared among receptors for multiple cytokines such as IL 2  [ **IL2** ] , IL 4, IL 7, IL 9 and IL 15.  Ref: 12069389 Am J Reprod Immunol, 2002
- The chicken B cell marker ChB6, caspase 1, inhibitor of apoptosis protein 1 IAP 1, interleukin 15 receptor alpha chain IL 15Ralpha, interleukin 2  [ **IL2** ]  receptor gamma chain  [ **IL2RG** ]  IL 2Rgamma, and immunoglobulin supfamily gene ZOV3, as physiological candidate genes for chicken immune response, were selected to investigate associations with antibody kinetics to SRBC and killed B. abortus.  Ref: 12872968 Poult Sci, 2003
- The high affinity human interleukin 7 IL 7 R is a heterodimeric complex consisting of the IL 7Ralpha and common interleukin 2  [ **IL2** ]  receptor gamma  [ **IL2RG** ]  IL 2Rgamma c chains.  Ref: 11858939 Cell Signal, 2002
- Although it blocks IL 2  [ **IL2** ]  mediated proliferation, MPA does not inhibit cell survival and Bcl x L up regulation by IL 2  [ **IL2** ]  or other cytokines whose receptors share the common gamma chain CD132  [ **IL2RG** ] .  Ref: 12193749 J Immunol, 2002
- We have also characterized the genomic structure of the murine Tslpr gene which shows that in addition to primary sequence homology, it shares a common genomic organization of coding exons with the murine IL 2  [ **IL2** ]  receptor common gamma chain  **Il2rg** .  Ref: 11891057 Gene, 2002
- SOCS 1 regulates signaling in response to IL 2  [ **IL2** ]  and other gamma c  [ **IL2RG** ]  dependent cytokines in peripheral T cells.  Ref: 12665516 J Biol Chem< MedlineTA>J Biol Chem, 2002
- Supporting this view were the substantially diminished levels of IL 2  [ **IL2** ] , CD25, CD122, and CD132  [ **IL2RG** ]  mRNA found in AGC10 containing cultures of PHA activated lymphocytes.  Ref: 12467977 Mol Biochem Parasitol< MedlineTA>Mol Biochem Parasitol, 2002
- Expression of the interleukin 2  [ **IL2** ]  receptor gamma  [ **IL2RG** ]  gene was greater at 1 00 pM than 25 pM biotin, mediating increased endocytosis of interleukin 2  [ **IL2** ]  in biotin supplemented medium.  Ref: 12388078 Am J Physiol Cell Physiol, 2003
- In particular, gene addition strategies for inherited severe combined immunodeficiencies SCID due to adenosine deaminase ADA deficiency or defects of the interleukin 2  [ **IL2** ]  receptor gamma chain  [ **IL2RG** ]  represent potentially curative strategies based on gene transfer into hematopoietic cells using recombinant retroviral vectors.  Ref: 12108973 Curr Gene Ther, 2002
